# Chemical simulation of hypoxia in donor cells improves development of somatic cell nuclear transfer‐derived embryos and increases abundance of transcripts related to glycolysis

**DOI:** 10.1002/mrd.23392

**Published:** 2020-06-17

**Authors:** Raissa F. Cecil, Paula R. Chen, Joshua A. Benne, Taylor K. Hord, Lee D. Spate, Melissa S. Samuel, Randall S. Prather

**Affiliations:** ^1^ Department of Animal Sciences University of Missouri Columbia Missouri

**Keywords:** cellular reprogramming, hypoxia inducible factor, metabolism, porcine, somatic cell nuclear transfer

## Abstract

To improve efficiency of somatic cell nuclear transfer (SCNT), it is necessary to modify differentiated donor cells to become more amendable for reprogramming by the oocyte cytoplasm. A key feature that distinguishes somatic/differentiated cells from embryonic/undifferentiated cells is cellular metabolism, with somatic cells using oxidative phosphorylation (OXPHOS) while embryonic cells utilize glycolysis. Inducing metabolic reprogramming in donor cells could improve SCNT efficiency by priming cells to become more embryonic in nature before SCNT hypoxia inducible factor 1‐α (HIF1‐α), a transcription factor that allows for cell survival in low oxygen, promotes a metabolic switch from OXPHOS to glycolysis. We hypothesized that chemically stabilizing HIF1‐α in donor cells by use of the hypoxia mimetic, cobalt chloride (CoCl_2_), would promote this metabolic switch in donor cells and subsequently improve the development of SCNT embryos. Donor cell treatment with 100 µM CoCl_2_ for 24 hr preceding SCNT upregulated messenfer RNA abundance of glycolytic enzymes, improved SCNT development to the blastocyst stage and quality, and affected gene expression in the blastocysts. After transferring blastocysts created from CoCl_2_‐treated donor cells to surrogates, healthy cloned piglets were produced. Therefore, shifting metabolism toward glycolysis in donor cells by CoCl_2_ treatment is a simple, economical way of improving the in vitro efficiency of SCNT and is capable of producing live animals.

## Graphical abstract

Hypoxic culture of donor cells used for porcine somatic cell nuclear transfer (SCNT) has previously been shown to induce cellular metabolic reprogramming and improve development of cloned embryos. In this study, treating donor cells with a hypoxia mimetic, cobalt chloride, further improved SCNT efficiency as compared with hypoxic culture. Therefore, cobalt chloride treatment can be used as a simple and economical alternative to hypoxic culture for porcine SCNT donor cells.

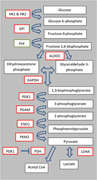

**Abbreviations**
BCL2: B‐cell leukemia/lymphoma 2
BNIP3: BCL2/adenovirus E1B 19 kDa protein‐interacting protein 3
CoCl_2_: cobalt chloride
COCs: cumulus‐oocyte complexes
HIF1‐α: hypoxia inducible factor 1‐α
HIFs: hypoxia inducible factors
LDHA: lactate dehydrogenase A
OXPHOS: oxidative phosphorylation
PDK1: pyruvate dehydrogenase kinase 1
PGAM1: phosphoglycerate mutase 1
PKM2: pyruvate kinase muscle isozyme M2
PVA: polyvinyl alcohol
SCNT: somatic cell nuclear transfer
TL‐Hepes: Tyrode's lactate 4‐(2‐hydroxyethyl)‐1‐piperazineethanesulfonic acid
TUNEL: terminal deoxynucleotidyl transferase (TdT) dUTP nick‐end labeling

## 1 INTRODUCTION

Since the birth of the first animal cloned with a somatic cell in 1996, somatic cell nuclear transfer (SCNT) has developed into a useful research tool (Wilmut, Schnieke, McWhir, Kind, & Campbell, 1997). Today SCNT is used for biomedical models, including xenotransplantation, as well as agricultural models that have led to the discovery of novel treatments for human diseases, animals that are disease resistant, and have put animal‐to‐human organ transplant within reach (Whitworth & Prather, 2017; Prather, Lorson, Ross, Whyte, & Walters, 2013). Even with the current success of SCNT‐created animals, the overall efficiency of SCNT remains low (<5%) with few live births resulting from the SCNT process (Whitworth & Prather, 2010). Due to the lack of authentic embryonic stem cells and induced pluripotent stem cell lines capable of producing live pigs, porcine SCNT is limited to the use of somatic cell types. Since somatic cells have already undergone some degree of differentiation, a possible explanation for poor SCNT efficiency is the inability to successfully remodel somatic nuclei through the SCNT process. A key feature that distinguishes embryonic/undifferentiated cells from somatic/differentiated cells is the metabolism that is used. Differentiated cells utilize mitochondrial oxidative phosphorylation (OXPHOS), while undifferentiated cells use glycolysis. There is mounting evidence to suggest that metabolic reprogramming, or the switch from OXPHOS to glycolysis, is necessary to revert cells back to an undifferentiated state and maintain stemness (Prigione et al., 2014).

HIFs are a class of master transcription factors responsible for the cellular survival response to hypoxic conditions. HIF stabilization promotes the transcription of target genes related to glycolysis, angiogenesis, cell survival and proliferation, cell migration, apoptosis, and erythropoiesis (Hu, Wang, Chodosh, Keith, & Simon, 2003). Hypoxic stress is alleviated by these downstream targets by modifying the need for oxygen for cellular mechanisms, such as energy production, or allowing for greater oxygen delivery. For example, downstream targets related to glucose metabolism, such as the glucose transporters *SLC2A1* and *SLC2A3*, allow for energy production through glycolysis as opposed to mitochondrial OXPHOS, which can only occur in the presence of oxygen (Semenza, 2000).

Previous studies have shown that donor cell culture in hypoxia (1.25% O_2_) results in an upregulation of genes related to glycolysis in donor cells, as well as increased blastocyst production and in utero survivability following SCNT (Mordhorst et al., 2018, 2019). However, hypoxic cell culture can be costly and often requires specialized mixed gas tanks to achieve low oxygen tensions. There is also no reliable way to monitor the oxygen tension that the donor cells are being exposed to when cultured in hypoxia, as it requires culture in chambers that must remain sealed. In addition, HIF 1‐α, the modulator of the hypoxic response in cells, has a high turnover rate with degradation occurring in 5–8 min once cells are exposed to atmospheric oxygen levels. During the SCNT process, the time between cell collection and cell‐oocyte fusion/activation is typically greater than 1 hr. Therefore, the influence of HIF 1‐α in these cells may be greatly diminished by the conclusion of the SCNT process.

Due to the possible instability of hypoxia inducible factor 1‐α (HIF1‐α) in hypoxia cultured cells, we proposed a chemical hypoxia mimetic that allows a sustained effect of HIF1‐α outside of physiological hypoxia. In normoxia, HIF1‐α is hydroxylated by prolyl hydroxylases that require oxygen and iron for their enzymatic activity. This hydroxylation serves as a docking site for Von Hippel Lindeau protein that marks HIF1‐α for degradation by the 26S proteasome. In hypoxic conditions, the oxygen required for the prolyl hydroxylases is not available; and therefore, the cascade of events leading to HIF1‐α degradation cannot be initiated. This allows HIF1‐α protein to accumulate in the cytoplasm and subsequently translocate to the nucleus to dimerize with HIF1‐β and direct transcription of downstream targets (Semenza, 2000). Cobalt chloride (CoCl_2_) is a known hypoxia mimetic that inhibits the activity of prolyl hydroxylases by replacing the required iron domain of the prolyl hydroxylases with cobalt (Hirsila et al., 2005). This chemical simulation allows stabilization of the volatile HIF1‐α, even in the presence of atmospheric oxygen. Once stabilized, HIF1‐α can activate its downstream targets including genes that induce the reprogramming of metabolic processes to favor glycolytic metabolism over OXPHOS.

Therefore, the objective of this study was to determine if treatment of somatic donor cells with the hypoxia mimetic, CoCl_2_, can induce metabolic reprogramming in the donor cells and promote better nuclear reprogramming before SCNT to improve development of SCNT embryos.

## 2 RESULTS

### 2.1 Impact of CoCl_2_ on cell viability

**Figure 1:**
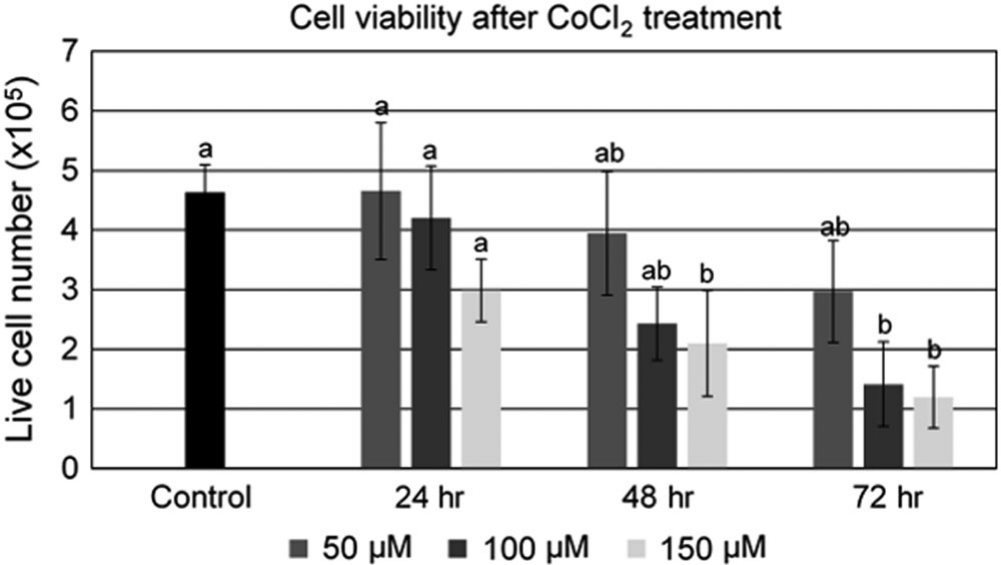
Cell viability after treatment with 0, 50, 100, or 150 µM of CoCl_2_ for 24, 48, or 72 hr. Data represented as means ± *SEM*. Statistical differences represented by different lowercase letters (a, b). *SEM*, standard error of mean

Cell number and viability was determined by Trypan blue exclusion after culture in 50, 100, or 150 µM of CoCl_2_ for 24, 48, or 72 hr (Figure 1). Live cell number was not different between any CoCl_2_ concentrations after 24 hr of culture. After 48 hr of culture, live cell number was significantly lower in the 150 µM treatment group as opposed to the 50 and 100 µM, or untreated cell groups. After 72 hr of culture, live cell number was negatively impacted in the 100 and 150 µM treatment groups as compared with the 50 µM and untreated groups.

**Figure 2:**
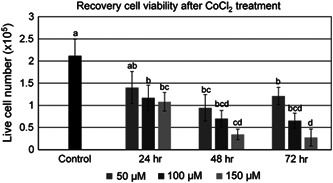
Cell viability following a 72 hr recovery period after treatment with 0, 50, 100, or 150 µM of CoCl_2_ for 24, 48, or 72 hr. Data represented as means ± *SEM*. Statistical differences represented by different lowercase letters (a, b, c, d). *SEM*, standard error of mean

Long‐term effects of CoCl_2_ treatment were determined by analysis of cell viability after a 3‐day recovery period following CoCl_2_ exposure (Figure 2). Only the 24‐hr 50 µM treatment of CoCl_2_ was capable of recovering cell viability to numbers comparable to the untreated control. The 50 µM treatment of CoCl_2_ did become detrimental to cell viability following 48 and 72 hr of exposure. The 100 µM CoCl_2_ treatment was comparable to the 50 µM treatment at all time points. The 150 µM treatment was significantly lower than the 50 µM treatment after 48 and 72 hr of CoCl_2_ exposure. Based on the results of these two studies, a treatment of 24‐hr exposure to 100 µM CoCl_2_ was chosen for the remainder of the study.

### 2.2 Gene expression in donor cells following CoCl_2_ exposure

Real‐time quantitative polymerase chain reaction (PCR) was used to analyze differences in message abundance between CoCl_2_ treated donor cells, hypoxia treated donor cells, and untreated control cells (Table 1) for HIF1‐α and non HIF1‐α gene targets (Liu, Shen, Zhoa, & Chen, 2012). Glucose transporters, *SLC2A1* and *SLC2A3*, as well as glycolytic enzymes *HK1*, *HK2*, *GPI*, *ALDOC*, *GAPDH*, *PGK1*, *PGAM1*, *ENO1*, *PKM2*, *PDK1*, and *LDHA* were upregulated in the CoCl_2_ group compared with the control. The same transcripts, with the exception of *SLC2A1, ALDOC, GAPDH*, and *PGAM1* were also upregulated in the hypoxia group compared with the control. Transcript abundance of the mitophagy‐associated gene *BNIP3*, *GPI*, and *PDK1* were differentially expressed between all treatment groups with the lowest expression present in the control cells and the highest expression in the CoCl_2_ cells. Non HIF1‐α targets, *TALDO1*, *EPAS1*, *YWHAG*, *LDHB*, and *BCL2* were not differentially expressed between the groups.

**Table 1:** Normalized abundance ± *SEM* of gene products related to glycolysis and mitophagy. Treatments include a control (cultured at 5% O_2_ for 3 days), CoCl_2_ treatment (100 µM CoCl_2_ for 24 hr), and a hypoxic treatment (cultured at 1% O_2_ for 3 days)

Gene name	Control	CoCl_2_	Hypoxia
*SLC2A1* *	1.88 ± 0.38^a^	3.25 ± 0.32^b^	2.29 ± 0.12^a,b^
*SLC2A3* *	1.61 ± 0.26^b^	3.25 ± 0.39^b^	3.75 ± 0.34^b^
*HK1* *	2.02 ± 0.17^a^	3.30 ± 0.25^b^	3.02 ± 0.18^b^
*HK2* *	10.59 ± 1.92^a^	22.36 ± 1.68^b^	19.24 ± 1.03^b^
*GAPDH* *	3.27 ± 0.34^a^	6.38 ± 0.49^b^	4.33 ± 0.44^b^
*PGK1* *	1.03 ± 0.10^a^	2.06 ± 0.09^b^	1.77 ± 0.10^b^
*ENO1* *	5.88 ± 0.44^a^	10.28 ± 0.68^b^	9.15 ± 0.97^b^
*PKM2* *	3.70 ± 0.25^a^	6.30 ± 0.59^b^	5.59 ± 0.48^b^
*PDK1* *	3.82 ± 0.48^a^	7.10 ± 0.05^b^	5.66 ± 0.51^c^
*LDHA* *	2.16 ± 0.22^a^	3.45 ± 0.28^b^	3.57 ± 0.26^b^
*LDHB*	0.11 ± 0.01	0.11 ± 0.001	0.12 ± 0.02
*BNIP3* *	2.02 ± 0.40^a^	5.54 ± 0.32^b^	3.69 ± 0.31^c^
*TALDO1*	0.81 ± 0.10	1.03 ± 0.09	0.85 ± 0.09
*EPAS1*	0.32 ± 0.07	0.55 ± 0.17	0.24 ± 0.04
*YWHAG*	0.39 ± 0.04	0.44 ± 0.04	0.39 ± 0.02
*BCL2*	0.55 ± 0.03	0.65 ± 0.07	0.53 ± 0.05

*Note*: ^a,b,c^Represent differences between treatments with *p* < .05 considered significant.

Abbreviation: *SEM*, standard error of mean.

* Indicates genes that are HIF targets.

**Table 2:** Blastocyst‐stage embryo development and quality parameters on Day 6 between embryos created from CoCl_2_ treated donor cells and control donor cells

Quality parameter	Control	CoCl_2_
Blastocyst rate (%) ± *SEM*	32.55 ± 1.87^a^	50.29 ± 2.57^b^
Total cell number ± *SEM*	38.99 ± 3.03^a^	51.96 ± 3.34^b^
% TUNEL positive ± *SEM*	7.04 ± 0.78	6.51 ± 0.72

*Note*: ^a,b^Represent differences between treatments with *p* < .05 considered significant.

Abbreviations: *SEM*, standard error of mean; TUNEL, terminal deoxynucleotidyl transferase dUTP nick‐end labeling.

### 2.3 SCNT embryo development and quality

**Figure 3:**
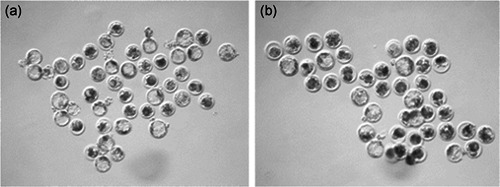
Representative images of blastocyst stage embryos created from (a) CoCl_2_ treated donor cells and (b) control donor cells

**Figure 4:**
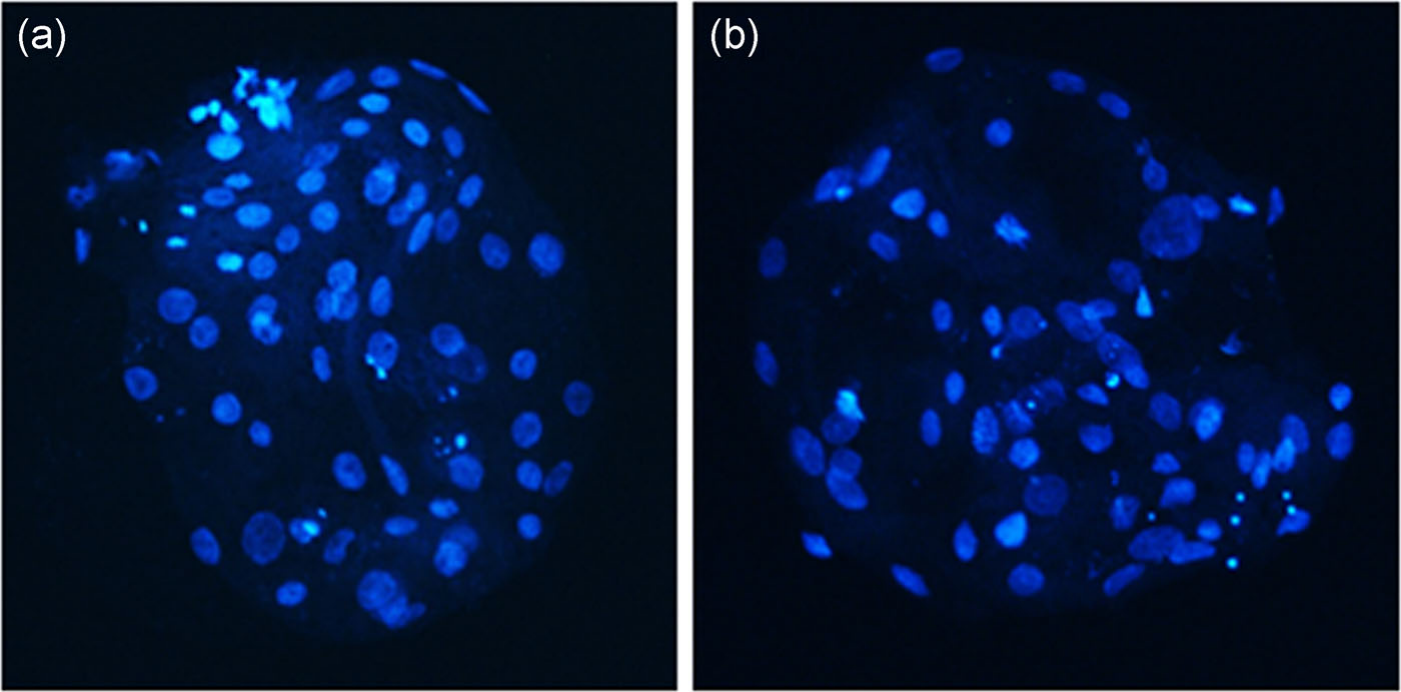
Representative images of Hoechst stained blastocyst stage embryos created from (a) CoCl_2_ treated donor cells and (b) control donor cells

The use of CoCl_2_‐treated donor cells for SCNT resulted in an increased rate of development to the blastocyst stage compared with untreated control donor cells (50.3 ± 2.6% vs. 32.6 ± 1.9%; *p* = .0002; Figure 3), as well as an increase in the total number of nuclei within the blastocyst‐stage embryos (52.0 ± 3.3 vs. 39.0 ± 3.0; *p* = .014; Figure 4). Evaluation of DNA damage by the terminal deoxynucleotidyl transferase dUTP nick‐end labeling (TUNEL) assay revealed no difference in the number of apoptotic nuclei between the groups (*p* = .64; Table 2).

### 2.4 Gene expression in SCNT blastocyst stage embryos produced by CoCl_2_ donor cells

Genes that were evaluated in donor cells were also analyzed in blastocyst‐stage embryos created with CoCl_2_ treated donor cells and blastocyst‐stage embryos created from untreated control cells (Table 3). Of the genes evaluated, *SLC2A1*, *PGAM1*, and *LDHA* were upregulated in Day 6 blastocyst‐stage embryos created from CoCl_2_ treated donor cells compared with control donor cells (*p* < .05).

**Table 3:** Normalized abundance ± *SEM* of gene products related to glycolysis and mitophagy. Treatments include Day 6 blastocyst stage embryos created from control donor cells and CoCl_2_ treated donor cells (100 µM CoCl_2_ for 24 hr)

Gene name	Control	CoCl_2_	*p* Value
*SLC2A1*	5.86 ± 0.66^a^	8.06 ± 0.44^b^	.0497
*SLC2A3*	2.05 ± 0.47	2.74 ± 0.16	.2370
*HK1*	0.12 ± 0.01	0.16 ± 0.03	.0978
*HK2*	24.30 ± 3.32	30.87 ± 2.68	.0989
*GPI*	0.66 ± 0.10	0.88 ± 0.04	.0917
*ALDOC*	0.37 ± 0.12	0.50 ± 0.03	.3755
*GAPDH*	2.45 ± 0.52	2.38 ± 0.50	.4626
*PGK1*	0.18 ± 0.02	0.25 ± 0.04	.0955
*PGAM1*	3.08 ± 0.10^a^	3.88 ± 0.26^b^	.0446
*ENO1*	1.31 ± 0.12	1.55 ± 0.09	.0916
*PKM2*	0.47 ± 0.11	0.66 ± 0.11	.1518
*PDK1*	2.26 ± 0.58	2.48 ± 0.56	.3997
*LDHA*	0.08 ± 0.01^a^	0.15 ± 0.02^b^	.0315
*BNIP3*	4.99 ± 0.66	6.76 ± 0.68	.1348
*TALDO1*	3.90 ± 0.45	4.58 ± 0.74	.2414
*YWHAG*	0.09 ± 0.01	0.13 ± 0.03	.1302
*BCL2*	4.49 ± 0.57	4.94 ± 0.69	.6432
*POU5F1*	476.97 ± 136.52	614.25 ± 35.49	.1928
*VEGFA*	2.99 ± 0.23	3.73 ± 0.53	.2678

*Note*: ^a,b^Represent differences between treatments with *p* < .05 considered significant.

Abbreviations: *SEM*, standard error of mean.

**Table 4:** Birthweights and status of piglets born from SCNT embryos created from CoCl_2_ treated donor cells

Piglet #	Birth weight, kg	Weaning weight, kg
1	0.845	4.120
2	1.155	4.720
3 (stillborn)	0.980	–
4 (stillborn)	0.800	–
5 (stillborn)	0.995	–
Avg	0.955	4.420

Abbreviation: SCNT, somatic cell nuclear transfer.

### 2.5 Cloned piglet production with CoCl_2_ treated donor cells

**Figure 5:**
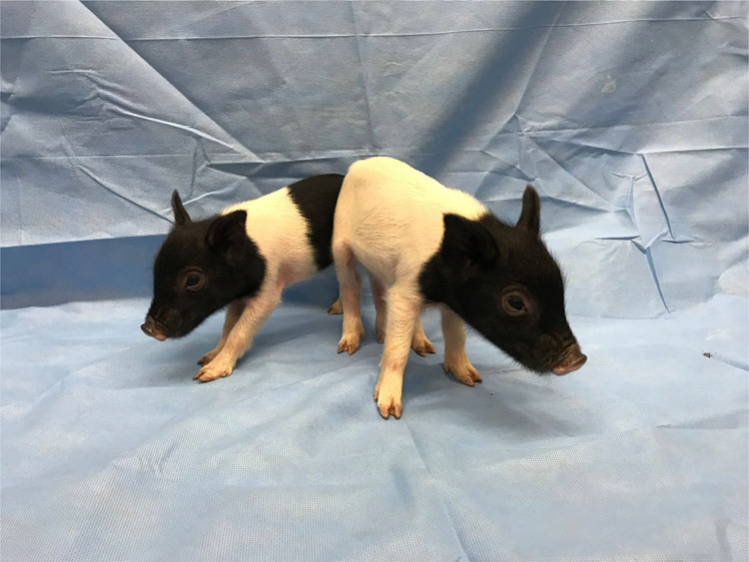
Images of cloned piglets produced from SCNT embryos created from CoCl_2_ treated donor cells. SCNT, somatic cell nuclear transfer

Following surgical embryo transfer to two recipient surrogates, both surrogates were confirmed pregnant by ultrasound at 25 and 38 days of gestation. At 52 days of gestation, one of the two surrogates had exhibited estrus and was no longer pregnant. At 120 days of gestation, the remaining pregnant surrogate farrowed naturally and delivered five piglets. Three of the five piglets were stillborn, and the surviving two piglets were healthy with no signs of abnormalities (Figure 5). No obvious defects were detected in the stillborn piglets; however, a necropsy was not performed. Birthweights ranged from 0.800 to 1.155 kg, with an average birthweight of 0.955 kg. Weaning weights recorded at 3 weeks were 4.720 and 4.120 kg, for an average weight of 4.420 kg (Table 4).

**Table 5:** RT‐PCR primers

Gene	Forward primer 5′ → 3′	Reverse primer 5′ → 3′	Accession #
YWHAG	TCCATCACTGAGGAAAACTGCTAA	TTTTTCCAACTCCGTGTTTCTCTA	XM_005661962.3
PKM2	ATGCAGTCTTGGATGGAGCTGACT	ATTGCAAATGGTAGATGGCGGCCT	AJ557236.1
SLC2A1	TCCACACCCACTTTGTCACACTGA	AGCCTCAACTCCCACATCACTGAA	XM_021096908.1
SLC2A3	CCCTCAGCTGCATTCTATTT	GTCTCAGGGACTTTGAAGAAG	XM_021092392.1
PGK1	CGCTTTCTGCATCTCCACTTGGCA	GCTGTGCAATGGTTCAAGGGTTCCT	NM_001099932.2
PDK1	ACCAGGACAGCCAATACAAGTGGT	ACGTGGACTTGAATAGGCGGGTAA	NM_001159608.1
TALDO1	TGAAGCGGCAGAGGATGGAGAGC	TCGTCGATGGCGTTGAAGTCGC	NM_001244935.1
EPAS1	AAGCAAAGACATGTCCACCGAGCG	GTGGCTGACTTGAGGTTGACGGTG	NM_001097420.1
HK1	TCTTGATCGACTTCACCAAGAGGG	TCGCTCTCGATCTGCGAGAGATACTT	NM_001243184.1
HK2	GAATTTGATGCGGCCGTGGATGAA	CCAGGTACATGCCGCTGATCATTT	NM_001122987.1
ENO1	TCGGAGTTCTACAGGTCGGGCAAG	TGGTCCGGTGAGATGTACCTGCTG	XM_021095280.1
PGAM1	CAGTGCTGGATGCCATTGACCAAA	GCTTGGCAGCAGTTTCTGCCTTAT	XM_003483535.4
LDHA	TTCAGCCCGGTTCCGTTACCTAAT	TTCTTCAGGGAGACACCAGCAACA	NM_001172363.2
LDHB	TAAGCATGGGCTTTGACTCTGGGA	ACTCCCGGCTTCTAGGTTGTAGTA	NM_001113287.1
VEGFA	CAAACCTCACCAAGGCCAGCACAT	CGAGCAAGGCCCACAGGGATTTTC	NM_214084.1
GPI	CCAGGAGACCATCACAAATG	TAGACAGGGCGACAAAGT	NM_214330.1
ALDOC	TCTTCCATGAGACCCTCTAC	TACACCCTTGTCCACCTT	NM_001243928.1
BNIP3	GGATTACATGGAGAGGAGGA	GTGCTTGAAGAGGAGGAAC	XM_003359404.4
BCL2	ACTGAATGCCCTCCGGTACC	ATCCCCATGGCTGCAGTGAA	XM_003130557.2
ACTB	TCTGGCACCACACCTTCT	TGATCTGGGTCATCTTCTCAC	DQ178122.1
POU5F1	TTTGGGAAGGTGTTCAGCCAAACG	TCGGTTCTCGATACTTGTCCGCTT	NM_001113060.1

Abbreviation: RT‐PCR, real‐time polymerase chain reaction.

## 3 DISCUSSION

**Figure 6:**
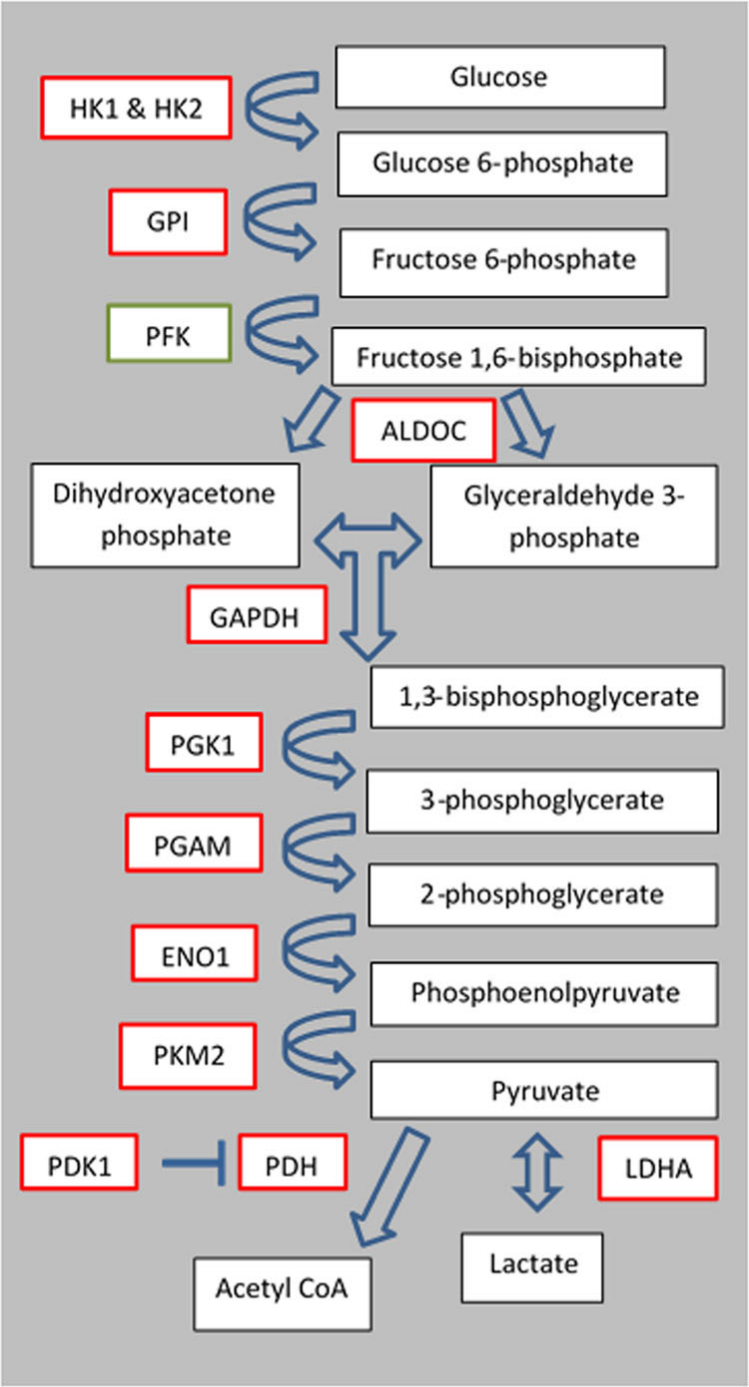
Schematic of glycolysis. Red rectangles represent enzyme transcripts that were differentially expressed between CoCl_2_ treated fibroblasts and control fibroblasts. Green rectangles represent enzyme transcripts that were not evaluated. ALDOC, aldolase C; CoA, coenzyme A; ENO1, enolase 1; HK1, hexokinase 1; GAPDH, glyceraldehyde 3‐phosphate dehydrogenase; GPI, glucose‐6‐phosphate isomerase; LDHA, lactate dehydrogenase A; PDH, pyruvate dehydrogenase; PDK1, pyruvate dehydrogenase kinase 1; PFK, phosphofructokinase; PGAM, phosphoglycerate mutase 1; PGK1, phosphoglycerate kinase 1; PKM2, pyruvate kinase muscle isozyme M2

The purpose of this study was to understand the effect of CoCl_2_ treatment on metabolism in SCNT donor cells and the resultant effect on SCNT efficiency in vitro with these donor cells. Analysis of HIF1‐α targets related to glycolysis and cell survival in donor cells cultured in either 5% O_2_ (control), 1% O_2_ (hypoxia), or 5% O_2_ with CoCl_2_ treatment was analyzed to understand the effect that HIF1‐α stabilization through physiological or chemical means had on gene expression (Table 5). Hypoxic culture of fibroblasts and fibroblasts cultured with CoCl_2_ resulted in an increase in messenger RNA (mRNA) abundance of glucose transporters *SLC2A1* and *SLC2A3*, as well as glycolysis‐related enzymes *HK1* and *HK2*, *GPI*, *ALDOC*, *GAPDH*, *PGK1*, *PGAM*, *ENO1*, *PKM2*, *PDK1*, and *LDHA*, all of which are HIF downstream targets. (Figure 6).

Although the majority of these enzymes are basic glycolytic enzymes that could indicate that an increase in glycolytic activity is occurring, enzymes, such as pyruvate kinase muscle isozyme M2 (PKM2), pyruvate dehydrogenase kinase 1 (PDK1), and lactate dehydrogenase A (LDHA) have unique roles that are specific to less differentiated cells, such as cancer cells, that are being pushed away from oxidative metabolism. Pyruvate kinase muscle isozyme M2 is one of the four isoforms of pyruvate kinase, produced by alternative splicing, and is specifically associated with proliferating cells and cancer cells (as reviewed by Dong et al., 2016). In the analysis of mRNA abundance of glycolytic enzymes associated with the Warburg effect, it was determined that blastocyst stage‐embryos exclusively expressed the fetal *PKM2* as opposed to the adult *PKM1* (Redel et al., 2011).

In an aerobic system, once pyruvate has been produced through glycolysis, it is subsequently converted to acetyl coenzyme A (CoA) through the mitochondrial enzyme pyruvate dehydrogenase. However, in glycolytic systems, the production of the enzyme PDK1 results in phosphorylation of pyruvate dehydrogenase which inactivates the complex and directs pyruvate away from the TCA cycle, inhibiting its oxidation. PDK1 has been demonstrated by microarray and chromatin immunoprecipitation to be a direct target of HIF1‐α, and is an important player in the switch from aerobic to anaerobic metabolism through its ability to block acetyl CoA production so that pyruvate can be converted to lactate (Kim, Tchernyshyov, Semenza, & Dang, 2006).

Since PDK1 increases availability of pyruvate in the cell, it is then able to be converted to lactate by LDHA. The conversion of pyruvate to lactate is crucial for anaerobic glycolysis. In human pancreatic cancer cells, *LDHA* is upregulated by hypoxia and is directly activated by HIF1‐α. Induced expression of LDHA promotes the proliferation and migration of pancreatic cancer cells, and knocked down expression inhibits cell growth and migration (Cui et al., 2017). This indicates that LDHA and its effect in hypoxic conditions is crucial for cancer cell survival.

Although the majority of gene expression changes found in this study relate to the SCNT donor cells, there were also several genes upregulated in CoCl_2_ treated donor cell SCNT blastocyst stage embryos (Table 3). Glucose transporter *SLC2A1*, and glycolytic enzymes *PGAM1* and *LDHA* were found to be upregulated in embryos created from CoCl_2_ treated donor cells as compared with those created from control donor cells. Although glucose is not a component of the embryo culture media used in this study, increased glucose uptake has been shown to be associated with improved embryo viability in bovine (Renard, Philippon, & Menezo, 1980), mouse (Gardner & Leese, 1987) and human (Gardner, Wale, Collins, & Lane, 2011) systems. Phosphoglycerate mutase 1 (PGAM1) enzymatic activity has been proposed as a potential alternative glycolytic pathway in rapidly proliferating cells that do not have increased pyruvate kinase activity. Phosphorylation of PGAM1 by the phosphate donor phosphoenolpyruvate, which is typically associated with PKM2 activity, promotes increased pyruvate production and allows for a higher glycolytic flux (Vander Heiden et al., 2010). LDHA promotes lactate production, and aligning with the Warburg effect, lactate production in the presence of oxygen is associated with rapidly proliferating cells. During blastocyst formation, there is a transition from the lactate dehydrogenase B isoform to the LDHA isoform which is associated with lactate production as opposed to pyruvate production (as reviewed by Krisher & Prather, 2012). Therefore, the upregulation of *LDHA* at the blastocyst stage in the embryos created from CoCl_2_ treated donor cells as compared with control SCNT embryos could indicate that a more natural gene expression profile in the blastocysts is promoted by metabolic reprogramming of CoCl_2_ treated donor cells before SCNT.

CoCl_2_ treatment of donor cells resulted in greater (~18% increase) blastocyst stage embryo development and improved embryo quality (13 more cells per blastocyst) as compared with control embryos (Table 2). However, previous studies have shown that analysis of blastocyst stage embryo qualities alone is not indicative of the in utero survival and live birth potential of embryos (Redel et al., 2016). To demonstrate that CoCl_2_ treatment of donor cells could result in the live birth of piglets following SCNT, embryo transfer was conducted. An untreated control donor cell comparison was not conducted in this study due to the number of animals that would need to be utilized, and surgeries that would have to be performed. The purpose of the embryo transfer was to ensure that there were no lethal effects of the donor cell treatment that would prevent the in utero survival of these embryos. Of the two surrogates used for embryo transfer, one was able to maintain pregnancy to term. This surrogate delivered five piglets unassisted. Of the five piglets that were delivered, three were stillborn. From outward visual inspection and birth weights, the three piglets did not have any obvious abnormalities that would have resulted in their death and had healthy birthweights for an Ossabaw breed (Table 4). Due to the lack of outward abnormalities in these stillborn piglets, along with the birth of two live piglets that proved there was no lethal effect of the CoCl_2_ treatment, postmortem necropsies were not conducted. The two surviving piglets had healthy birth weights and weaning weights and have had no issues since their birth. Therefore, the birth of healthy clones from this experiment indicates that CoCl_2_ treatment of donor cells results in SCNT embryos that are capable of producing piglets and can be used as a viable option for future cloning studies.

Our findings indicate that the use of CoCl_2_ as a novel treatment for SCNT donor cells induces the same glycolytic response as culture in 1% oxygen (hypoxia) for 3 days. The use of the hypoxia mimetic allows the cells to be maintained in any oxygen tension, without the need for specialized gas tanks or chambers and eliminates the need for long term culture of donor cells in hypoxic conditions to establish the same effect. The upregulation of genes that are known to be downstream targets of HIF1‐α in the CoCl_2_ treated and hypoxia treated donor cells, along with the lack of differential expression of non‐HIF1‐α targets suggests that the transcription factor may be activated through these treatments. Therefore, promoting metabolic reprogramming in donor cells through CoCl_2_ treatment improves the efficiency of the SCNT process through alterations in gene expression in donor cells and resultant SCNT blastocysts, improvement in the quality and development rate of SCNT embryos, and production of healthy, cloned piglets.

## 4 MATERIALS AND METHODS

### 4.1 Ethics statement

Collection of ovaries from prepubertal gilts and use of live animals were in accordance with approved protocol and standard operating procedures by the Animal Care and Use Committee of the University of Missouri.

### 4.2 Determining optimal CoCl_2_ concentration

Dorsal tissue of gestational Day 35 wild‐type fetuses was removed and dissociated. Cells were cryopreserved in 500 µl aliquots and stored in liquid nitrogen until needed. Cells were thawed and cultured in Dulbecco's modified Eagle's medium (1 g/L glucose with phenol red) supplemented with 15% fetal bovine serum (Corning, Corning, NY) for 4 days in T25 flasks (Corning). For determining the working CoCl_2_ concentration, cobalt chloride hexahydrate (C8661; Thermo Fisher Scientific, Waltham, Massachusetts) was mixed fresh daily for each use. To achieve a 10 mM concentration of CoCl_2_, 11.89 mg of CoCl_2_ was dissolved into 5 ml MilliQ H_2_O. The solution was then added at a 1:100 µl ratio to culture media to achieve a final concentration of 100 µM. To evaluate the effect of increased CoCl_2_ concentrations on cell viability, cells were plated at equal density of 7.5 × 10^4^ cells/flask and the CoCl_2_ solution was added at 50, 100, and 150 µM. All concentrations were applied to cells for 24, 48, or 72 hr. Control cells were left untreated. After the 72 hr, CoCl_2_ treated and control cells were trypsinized and Trypan blue exclusion was used to determine live and total cell number. To evaluate the recovery ability of cells after CoCl_2_ exposure, the beforementioned conditions were applied to cells plated at equal densities, followed by aspiration of media containing CoCl_2_ and replacement with fresh media. The cells were grown for 3 days subsequent to CoCl_2_ removal and then trypsinized and subjected to Trypan blue exclusion to determine live and total cell number.

For SCNT, fibroblast cells were thawed 4 days before SCNT, counted by Trypan blue exclusion, plated at a density of 7.5 × 10^4^ cells/T25 flask and cultured in a humidified incubator with an atmosphere of 5% CO_2_, 5% O_2_, and 90% N_2_ at 37.5°C. On Day 3, 24 hr before SCNT, CoCl_2_ was added at a 100 µM concentration. The control cells were left untreated.

### 4.3 Oocyte collection and somatic cell nuclear transfer

Ovaries from a local abattoir (Smithfield, Milan, MO) were harvested and 18‐gauge needles attached to disposable 10 ml syringes were used to aspirate follicles that were 3–6 mm in size and showed normal morphology. Cumulus‐oocyte complexs (COCs) in follicular fluid were washed three times in Tyrode's lactate 4‐(2‐hydroxyethyl)‐1‐piperazineethanesulfonic acid (TL‐Hepes) before being placed in 100 mm polystyrene petri dishes. COC's displaying uniform cytoplasm and at least three layers of cumulus cells were selected and placed in maturation medium (TCM‐199 medium supplemented with 0.1% polyvinyl alcohol [PVA], 3.05 mM *d*‐glucose, 0.91 mM sodium pyruvate, 10 μg/ml of gentamicin, 0.57 mM cysteine, 10 ng/ml of EGF, 0.5 μg/ml of FSH, 0.5 μg/ml of LH, 40 ng/ml FGF2, 20 ng/ml LIF, and 20 ng/ml IGF1; Yuan et al., 2017) for 42–44 hr in a humidified incubator with an atmosphere of 5% CO_2_ in air at 37.5°C. Cumulus cells were stripped from oocytes by gentle vortex for 3 min in 0.1% (wt/vol) hyaluronidase in TL‐HEPES‐buffered saline with 0.1% PVA. Metaphase II oocytes were selected based on the presence of an extruded first polar body in the perivitelline space.

Metaphase II oocytes were placed on the stage of an inverted microscope equipped with micromanipulators in drops containing manipulation medium (Lai & Prather, 2004) supplemented with 7.0 µg/ml cytochalasin B. A hand‐tooled glass pipette was used to remove the polar body, and approximately 10% of the adjacent cytoplasm (presumably containing the metaphase plate). Following enucleation, a fibroblast cell was injected into the perivitelline space and pressed against the cytoplasm. Donor cells were then trypsinized and harvested for SCNT, with CoCl_2_ treated cells resuspended in manipulation medium containing 7.0 µg/ml cytochalasin B and 100 µM CoCl_2_. While injecting CoCl_2_ treated cells, 100 µM CoCl_2_ was present in the micromanipulation drops to sustain the treatment effect and prohibit HIF1‐α degradation. Oocyte‐donor cell couplets were then fused in fusion medium (0.3 M mannitol, 0.1 mM CaCl_2_, 0.1 mM MgCl_2_, 0.5 mM HEPES buffer, pH 7.2) by two direct current pulses (1‐s interval) at 1.2 kV/cm for 30 µs by using a BTX Electro Cell Manipulator (Harvard Apparatus, Holliston, MA). At least 1 hour after fusion, reconstructed embryos were fully activated for 30 min with 200 µM *N*,*N*,*N*′,*N*′‐tetrakis (2‐pyridylmethyl) ethane‐1,2‐diamine (Lee et al., 2015) in TL‐HEPES. Embryos were then incubated in MU‐2 media with 0.5 µM of histone deacetylase inhibitor Scriptaid, for 14–16 hr in a 5% carbon dioxide (atmospheric oxygen) incubator (Whitworth, Zhao, Spate, Li, & Prather, 2011; Zhao et al., 2009). The following morning, embryos were removed from Scriptaid treatment, washed, and placed in fresh MU‐2 media and cultured in an incubator with a humidified atmosphere of 5% CO_2_, 5% O_2_, and 90% N_2_ at 37.5°C until Day 6 post‐activation.

### 4.4 Blastocyst quality evaluation

Day 6 blastocyst‐stage embryos collected in pools of 15–25 per treatment were fixed in 4% paraformaldehyde in TL‐HEPES for 20 min, followed by permeabilization with 0.1% Triton X‐100 for 30 min. To assess DNA damage, blastocyst stage embryos were incubated with TUNEL stain for 30 min, and then Hoechst nuclear stain (10 µg/ml) for 5 min. Blastocyst‐stage embryos were visualized at 20x magnification on a microscope equipped with epi‐fluorescence, and total cells and TUNEL positive cells were quantified. The ratio of TUNEL positive cells to total cells was calculated to determine a percentage of DNA damaged cells per blastocyst‐stage embryo.

### 4.5 RNA extraction and complementary DNA synthesis

To evaluate gene expression in donor cells, cells were subjected to either CoCl_2_ treatment, hypoxic treatment, or left untreated. For all treatments, cells were plated at equal densities in T25 plates. For CoCl_2_ treatment, cells were maintained at 5% CO_2_, 5% O_2_, and 90% N_2_ at 37.5°C and 100 µM CoCl_2_ was added on the third day of culture, 24 hr before cell collection. For the hypoxic treatment, cells were placed in an incubator maintained at 5% CO_2_, 5% O_2_, and 90% N_2_ at 37.5°C for at least 4 hours before being transferred to a hypoxic chamber (Billups‐Rothenburg, San Diego, CA) supplemented with a 100 mm petri dish of milliQ H_2_O. The chamber was sealed and gassed for 2 min with 1% O_2_ using a mixed gas LiquidGas tank (1% O_2_, 5% CO_2_). The chamber was then placed back into the incubator at 5% CO_2_, 5% O_2_, and 90% N_2_ at 37.5°C and were left to grow for 3 days following hypoxic exposure.

Day 6 blastocyst‐stage embryos created with either CoCl_2_ treated donor cells or control donor cells were collected in pools of 35–50 and washed in diethyl pyrocarbonate‐treated phosphate‐buffered saline before being snap‐frozen in liquid nitrogen for storage at −80°C. Fibroblast cells cultured in CoCl_2_ for 24 hr, untreated, or cultured in 1% hypoxia for 3 days were trypsinized, pelleted, and snap‐frozen in liquid nitrogen for storage at −80°C. Three biological replicates were collected for each treatment. For blastocyst‐stage embryos, total RNA was extracted by using an RNeasy Micro Kit (Qiagen, Germantown, MD) and eluted in 12 μl of nuclease‐free water. All 12 μL of eluted RNA was used for complementary DNA (cDNA) synthesis by the SuperScript VILO cDNA Synthesis Kit (11754050; Thermo Fisher Scientific). For fibroblast cells, total RNA was extracted by using an RNeasy Mini Kit (Qiagen, Germantown, MD) and eluted in 30 µl of nuclease‐free water. RNA content was determined by using a Nanodrop 1000 Spectrophotometer (Thermo Fisher Scientific), and an appropriate amount of eluted RNA was added accordingly for cDNA synthesis by the SuperScript VILO cDNA Synthesis Kit (11754050; Thermo Fisher Scientific).

### 4.6 Relative quantitative PCR

Relative quantitative PCR was performed with each sample from cDNA synthesis. Message evaluated included HIF1‐α targets associated with glycolysis, autophagy, and pluripotency in fibroblast cells and blastocyst stage embryos (Table 5). Samples from each biological replicate were diluted to 5 ng/µL, and quantitative PCR was run in triplicate to determine differential expression of the selected transcripts with the conditions: 95°C for 3 min, and 40 cycles of 95°C for 10 s, 55°C for 10 s, and 72°C for 30 s. A dissociation curve was generated after amplification to ensure that a single product was amplified. Abundance of each mRNA transcript was calculated relative to a housekeeping gene, β actin, and a pig genome reference sample. The comparative quantification cycle method was used to determine relative mRNA expression for each treatment.

### 4.7 Surgical embryo transfer

For the embryo transfer experiment, donor cells used for SCNT were a wild‐type Ossabaw cell line (RRID NSRRC:0008) that had been proven clonable (Mordhorst et al., 2019). Following SCNT, Day 6 blastocyst‐stage embryos created from CoCl_2_‐treated donor cells were transferred into recipient surrogates. Briefly, two gilts 4 days post‐observed estrus were aseptically prepared for surgery, and the infundibulum was exposed by entry though the lower abdominal wall. A Tomcat catheter containing 42 blastocyst‐stage embryos was inserted into one ampullary‐isthmic junction of each surrogate where the blastocysts were deposited. Pregnancy was determined by ultrasound on Day 25 and monitored by biweekly ultrasounds thereafter. After farrowing, birth weights, weaning weights, and phenotypes were recorded.

## CONFLICT OF INTERESTS

The authors declare that there are no conflict of interests.
